# Endoscopic Endonasal Surgery of a Large Vidian Nerve Schwannoma With Preparation for Avoiding Major Vascular Injury

**DOI:** 10.7759/cureus.14230

**Published:** 2021-03-31

**Authors:** Chisato Tanaka, Masahiro Kikuchi, Mami Matsunaga, Koichi Omori, Takayuki Nakagawa

**Affiliations:** 1 Otolaryngology, Head and Neck Surgery, Osaka Red Cross Hospital, Osaka, JPN; 2 Otolaryngology, Head and Neck Surgery, Graduate School of Medicine, Kyoto University, Kyoto, JPN

**Keywords:** vidian nerve, schwannoma, endoscopic endonasal surgery, ultrasonic surgical aspirator, surgical navigation system

## Abstract

Vidian nerve schwannomas are extremely rare, and their surgical management requires an awareness of the surrounding vascular and nervous systems, including the internal carotid artery. Herein, we report a case of a vidian nerve schwannoma that was successfully removed using an endoscopic endonasal approach in a 21-year-old patient who presented with lacrimal hyposecretion. Imaging revealed a large mass extending to the middle cranial fossa posteriorly, to the pterygopalatine fossa laterally, and to the sphenoid sinus medially. The paraclival and petrosal portions of the internal carotid artery were displaced posteriorly. Endoscopic observation of the right nose demonstrated anterior displacement of the inferior portion of the middle turbinate. Based on the above, we suspected a vidian nerve schwannoma, and endoscopic endonasal surgery was performed with particular attention to avoid vascular injuries. An endoscopic transmaxillary approach was used to expose the anterior surface of the tumor. After confirming the pathological diagnosis intraoperatively, intracapsular resection of the tumor was completed using an ultrasonic surgical aspirator with Doppler monitoring of the location of the internal carotid artery. Endoscopic management of the surgical field and preparation to avoid vascular injury are essential for safe and efficient tumor resection.

## Introduction

The vidian nerve consists of sympathetic fibers originating from the deep petrosal nerve and parasympathetic fibers from the greater petrosal nerve which innervates the lacrimal gland and the nasopalatine mucosa. This nerve exits the cranial cavity anterior to the horizontal segment of the petrous internal carotid artery (ICA), courses anteriorly through the pterygoid canal, and exits the skull base through the foramen on the face of the pterygoid plates within the pterygopalatine fossa [1]. Therefore, the vidian nerve has emerged as a surgical landmark for the foramen lacerum and second genu of the ICA during endoscopic endonasal skull base surgery [2].

Schwannomas are benign tumors of the Schwann cells that coat the nerve sheath. Vestibular nerve schwannomas are the most common, followed by trigeminal nerve schwannomas [3]. Vidian nerve schwannomas are extremely rare. Herein, we present a case of a large vidian nerve schwannoma primarily manifesting with lacrimal hyposecretion that was successfully treated with endoscopic endonasal surgery.

## Case presentation

A previously healthy 21-year-old woman was referred to our hospital with an incidental finding of a right middle cranial fossa tumor measuring 44 mm in maximum diameter on computed tomography (CT) imaging performed for head injury. The patient had a six-year history of dryness affecting her right eye but without visual symptoms, nasal symptoms, or facial numbness.

**Figure 1:**
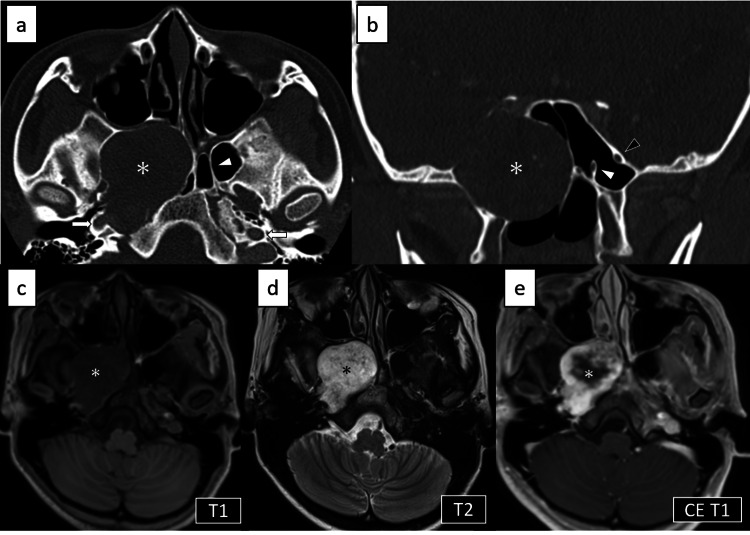
Preoperative CT and MRI The tumor occupied the floor of the sphenoid sinus and displaced the pterygopalatine fossa anteriorly on the right side (asterisk). a) Axial plain CT image. Vidian canal and foramen lacerum on the right side were not detected. White arrowhead: left vidian canal. White arrows: carotid canals. b) Coronal plain CT image. The surrounding bone was thinned, but no bony invasion was present. We were able to identify the vidian canal and foramen rotundum on the left side but not on the right side. White arrowhead: left vidian canal. Black arrowhead: left foramen rotundum. On MRI, the tumor was (c) low signal on T1-weighted image, (d) high signal on T2-weighted image, and (e) slightly and heterogeneously enhanced.

The Schirmer’s test was performed to assess tear secretion; the results were 6 mm on the right and 24 mm on the left, indicating decreased tear production on the right side. Nasal endoscopy showed anterior displacement of the posteroinferior part of the middle turbinate, but we were unable to visualize the tumor. Therefore, the patient underwent contrast-enhanced CT imaging, which revealed a slightly enhanced expansile soft tissue mass that occupied the floor of the sphenoid sinus and displaced the pterygopalatine fossa anteriorly on the right side with no bony invasion. The foramen rotundum, vidian canal, and foramen lacerum on the right side were not detected (Figure 1).

**Figure 2:**
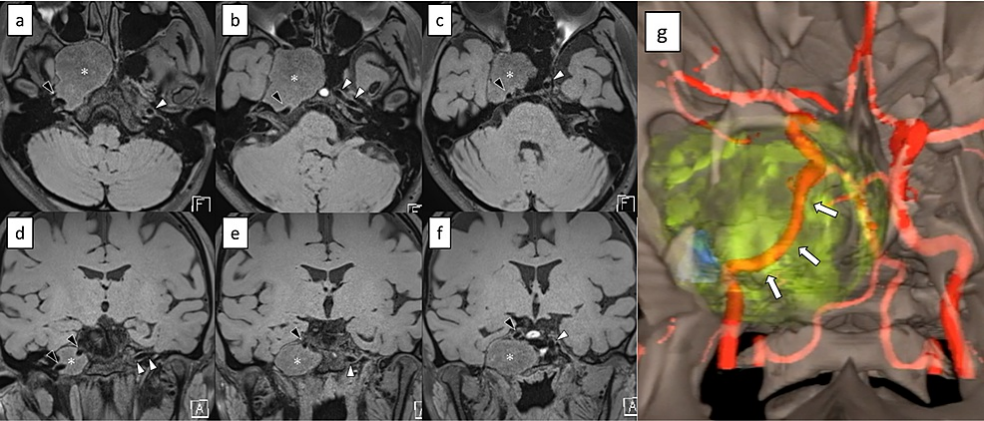
Preoperative assessment of the course of the internal carotid artery (a-f) Non-contrast MRI with DANTE-SPACE sequence. a) to c) are axial images (caudal to cephalad). d) to f) are coronal images (posterior to anterior). The internal carotid artery behind the tumor was appreciated by black blood image. g) Virtual image made from MRA and MRI using the surgical navigation system. Red: artery; yellow: tumor; blue: foramen ovale. The paraclival and petrous portion of the internal carotid artery on the right side was postero-laterally displaced by the tumor (arrows). Asterisk: tumor. Black arrowhead: internal carotid artery on the right side. White arrowhead: internal carotid artery on the left side.

On magnetic resonance imaging (MRI), the tumor had low signal intensity on T1-weighted images and high signal intensity on T2-weighted images, with slight and heterogeneous gadolinium enhancement (Figure 1). The course of the ICA was thoroughly evaluated using MRI and magnetic resonance angiography (MRA). MRI with the DANTE-SPACE sequence revealed that the paraclival and petrous portion of the right ICA were in contact with the tumor posteriorly, and its course was postero-laterally displaced by the tumor (Figure 2). Preoperative virtual images from MRA and MRI using the surgical navigation system (StealthStation™ ENT Navigation System, Medtronic, Minneapolis, MN, USA) were obtained in advance to evaluate the relationship between the tumor and the course of the adjacent ICA (Figure 2).

Based on imaging findings, the differential diagnoses were neurogenic tumor of vidian nerve (such as schwannoma and neurofibroma), chordoma or juvenile angiofibroma. However, juvenile angiofibroma was less likely due to the mild contrast effects on CT and MRI. Therefore, we suspected a neurogenic tumor of vidian nerve, which correlated with the patient’s clinical symptoms. However, as we were unable to visualize the tumor surface in the nasal cavity, preoperative biopsy was not possible, and the patient provided consent for surgery.

First, right endoscopic modified medial maxillectomy (EMMM) was performed to access the posterior wall of the maxillary sinus while preserving the nasolacrimal duct [4]. To secure a wide surgical field, the posterior base of the right inferior turbinate was dissected and retracted anteriorly, and the lower half of the right middle turbinate was resected and removed. The inferior turbinate was preserved and repositioned after completion of the surgery. During dissection of the posterior end of the middle turbinate, there was active bleeding from a branch of the sphenopalatine artery, which was cauterized with bipolar cautery. After removing the posterior wall of the maxillary sinus, the right descending palatine artery and the sphenopalatine artery were cauterized and transected by bipolar cautery, and the proximal maxillary artery was ligated with endoscopic liga-clips and transected. Then, the contents of the pterygopalatine fossa were retracted laterally to expose the tumor capsule. The capsule was resected and a small sample was obtained for frozen section, which confirmed a diagnosis of schwannoma.

**Figure 3:**
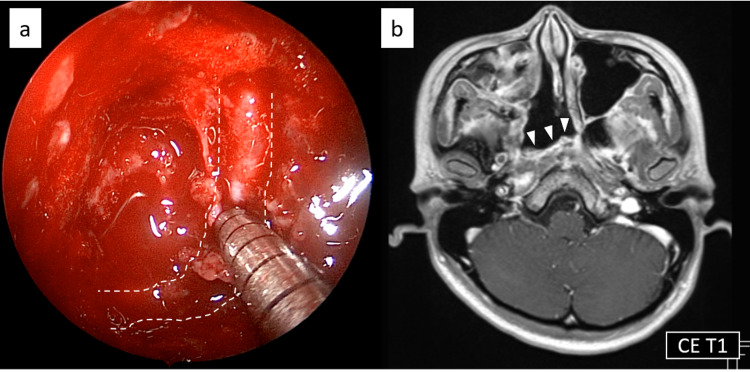
Intraoperative image and postoperative MRI a) Endoscopic image at the end of the surgery. The paraclival portion of the internal carotid artery was exposed. White dotted line shows the course of the internal carotid artery. b) Contrast-enhanced MRI five months after the surgery showed no apparent recurrence. White arrow heads: the pedicled nasal septum flap covering the exposed internal carotid artery.

After harvesting the pedicled nasal septal mucosal flap on the left side, ala of the vomer, and posterior part of the perpendicular plate of the ethmoid bone, both sides of the anterior walls of the sphenoid bone were removed to make a sufficient corridor for bi-nostril 2-surgeon/3- or 4-handed technique in case of active bleeding. Thereafter, intracapsular piecemeal tumor resection was started with a surgical navigation system following the course of the ICA in order to avoid major vascular injury. After the tumor was surgically decompressed, the residual tumor was crushed and aspirated using an ultrasonic surgical aspirator. Macroscopically, the tumor was highly vascular and bled during the resection. A Doppler ultrasound was used to identify the surrounding ICA intraoperatively. When the tumor was completely removed except for the capsule, the paraclival ICA was finally exposed (Figure 3), but no active bleeding or cerebrospinal fluid leak was observed. The exposed ICA was covered with a pedicled nasal septum flap, which was harvested from the left side in advance to prevent the possibility of a fatal rupture in the future. During the operation, the foramen lacerum and the pterygoid canal artery and nerve could not be visualized.

**Figure 4:**
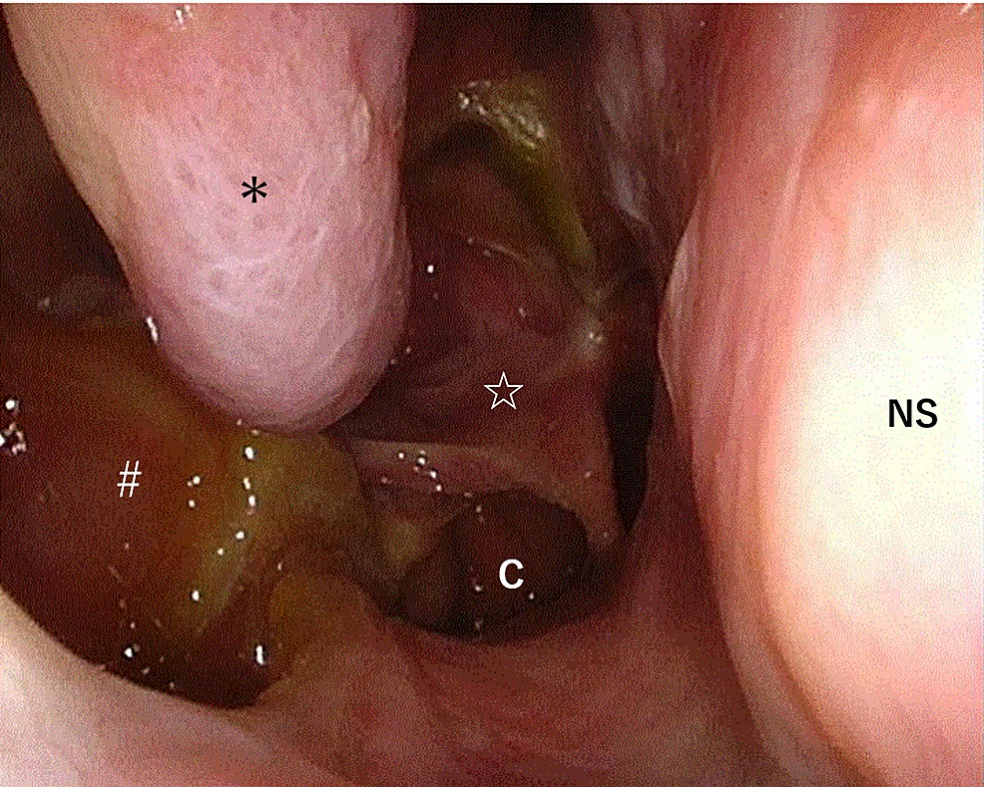
Endoscopic image 11 months after surgery. Preserved inferior turbinate (＊), opened pterygopalatine fossa (#) on the right side and pedicled nasal septum flap harvested from the left side (☆) can be identified. NS: nasal septum, C: choana.

The patient’s postoperative course was uneventful, with no bleeding or cerebrospinal fluid leaks, and she did not require a blood transfusion. She reported no change in cheek sensation and no worsening of right eye dryness. We did not insert gauze, but packed absorbable hemostat into the nose. Nasal irrigation with saline solution was started five days after the surgery and the patient was discharged from the hospital 15 days after the surgery. Post-operative MRI performed five months after the surgery showed no apparent tumor recurrence (Figure 3). Endoscopic image 11 months after surgery was shown in Figure 4.

On histological examination, the tumor was composed of spindle cells with comma-like nuclei. Secondary degenerative changes, including hyalinization, hemorrhage, and calcification, were observed. These findings confirmed the diagnosis of a schwannoma.

Although we did not observe the pterygoid canal during the surgery, and therefore could not confirm the continuity between the tumor and the vidian nerve, the patient’s clinical presentation and postoperative findings indicated that the origin of the schwannoma was the vidian nerve on the right side.

## Discussion

Head and neck are common sites for schwannomas, accounting for 25%-45% of all schwannomas. In contrast, vidian nerve schwannomas are extremely rare. To the best of our knowledge, only 10 such cases (including the current case) have been reported in the literature and their clinical features are summarized in Table 1 [1,3,5-10].

**Table 1:** Characteristics of previous case reports EES: Endoscopic endonasal surgery; CSF: Cerebrospinal fluid; F: Female; M: Male; NA: Not available.

No	Author	Age	Sex	Presentation	Tumor size	Treatment	Outcomes
1	Cheong et al., 2006 [5]	13	F	Headache with unilateral facial nerve palsy	12mm × 10mm	EES	N.A.
2	Honda et al., 2008 [6]	49	F	Unilateral hearing loss with serous otitis media	N.A.	Maxillary swing with endoscopic assistance	No recurrence (1 month)
3	Hackman et al., 2011 [1]	49	M	Occipital headache	10mm × 14mm	Follow up	N.A.
4	Hackman et al., 2011 [1]	58	M	Unilateral palate pain and lip numbness	N.A.	EES	N.A.
5	Wu et al., 2012 [7]	78	F	Unilateral oculomotor palsy with CSF leakage	N.A.	EES	No recurrence (24 months)
6	Hong et al., 2014 [8]	41	M	Occipital headache	28mm × 16mm	EES	N.A.
7	Yamasaki et al., 2015 [9]	49	F	Asymptomatic	20mm × 14mm	Radiation therapy	N.A.
8	Fortes et al., 2019 [3]	60	F	Unilateral facial hypoesthesia	N.A.	EES	No recurrence (3 months)
9	Masroor et al., 2018 [10]	54	F	Periorbital pressure, third cranial nerve palsy, visual field defects	41mm × 36mm	EES	No recurrence (28 months)
10	Tanaka et al., 2021 (current case)	21	F	Lacrimal hyposecretion	44mm × 36mm	EES	No recurrence (5 months)

Clinically, the patient described in the current report presented with decreased lacrimation, which is consistent with the innervation of the lacrimal gland by the vidian nerve. Surprisingly, this is the first case to preoperatively present with symptoms of vidian nerve dysfunction.

Endoscopic endonasal surgery was the most common surgical approach for vidian nerve schwannomas (seven out of 10 cases including the current case) [1,3,5,7,8,10]. During endoscopic endonasal surgery for removing vidian nerve schwannoma, the operation field, which is usually called "corridor" by skull base surgeons, was created depending on the size and extent of the tumor, but it is usually difficult to resect tumors using the trans-sphenoidal approach alone. Most tumors reported in the literature were removed in a wide surgical field after unilateral or bilateral wide-sphenoidectomy combined with trans-maxillary or trans-ethmoidal approaches. To the best of our knowledge, the tumor diameter in the current case is the largest reported to date. When performing an endoscopic endonasal resection of such a large tumor, management of intraoperative bleeding is important, and the preoperative assessment of the course of large vessels is necessary. In addition, the risk of postoperative rapture should also be considered in reconstruction at the end of the surgery. In the present case, invasive preoperative angiography was not performed. Instead, the course of the ICA was thoroughly evaluated to prevent fatal bleeding. The DANTE-SPACE sequence can provide complete blood suppression and black blood imaging, and MRI performed with this sequence is usually used for the assessment of the arterial vessel wall and plaques [11]. In the current case, this allowed us to clearly visualize the course of the ICA behind the tumor. Owing to the preoperative meticulous evaluation of the course of the ICA and the intraoperative use of Doppler ultrasound, we could safely remove the tumor around the ICA.

Intraoperative bleeding can be managed by packing gauze soaked with epinephrine or using bipolar cautery. When dissecting the middle turbinate, active bleeding was observed from a branch of the sphenopalatine artery. Although there were no complications caused by bleeding, on further consideration, we could have ligated the maxillary artery prior to resecting the middle turbinate as this may have reduced the amount of bleeding.

Compared to extracapsular resection, intracapsular resection is thought to be superior for the preservation of origin nerve function [12]. In this case, intracapsular piecemeal tumor resection was performed, and at the end of the surgery, the residual tumor was crushed and aspirated using an ultrasonic surgical device. Intracapsular tumor resection using an ultrasonic surgical device is thought to be safe and effective, especially for large tumors located near large vessels such as the ICA.

## Conclusions

We report a case of endoscopic endonasal surgery for a vidian nerve schwannoma that had invaded the pterygopalatine fossa and middle cranial fossa. By using an intraoperative navigation system, Doppler ultrasound, and an ultrasonic surgical device, we were able to perform minimally invasive surgery with complete intracapsular resection of the tumor.

## References

[1] Hackman T, Rickert CG, Getz AE, Uppaluri R (2011). Endoscopic surgical management of vidian nerve schwannoma. Laryngoscope.

[2] Kassam AB, Vescan AD, Carrau RL (2008). Expanded endonasal approach: vidian canal as a landmark to the petrous internal carotid artery. J Neurosurg.

[3] Fortes B, Beer-Furlan A, Balsalobre L, Vellutini E, Stamm A (2019). Endoscopic endonasal access for the treatment of Vidian nerve schwannoma: a case report. Braz J Otorhinolaryngol.

[4] Suzuki M, Nakamura Y, Nakayama M, Inagaki A, Murakami S, Takemura K, Yokota M (2011). Modified transnasal endoscopic medial maxillectomy with medial shift of preserved inferior turbinate and nasolacrimal duct. Laryngoscope.

[5] Cheong JH, Kim JM, Bak KH, Kim CH, Oh YH, Park DW (2006). Bilateral vidian nerve schwannomas associated with facial palsy: case report and review of the literature. J Neurosurg.

[6] Honda K, Asato R, Tanaka S, Endo T, Nishimura K, Ito J (2008). Vidian nerve schwannoma with middle cranial fossa extension resected via a maxillary swing approach. Head Neck.

[7] Wu SW, Chen WL, Chen WL, Chen MK (2012). Transnasal endoscopic resection of vidian nerve schwannoma accompanied by sphenoid mucopyocele and oculomotor palsy: a case report. B-ENT.

[8] Hong HP, Yoon SW, Park MJ, Jung SC (2014). A case of vidian nerve schwannoma: resection by endoscopic sinus surgery. Korean J Otorhinolaryngol-Head Neck Surg.

[9] Yamasaki A, Sedaghat AR, Lin GC, Curry WT, Shih HA, Gray ST (2015). A rare finding of schwannoma of the vidian canal: a case report. J Neurol Surg Rep.

[10] Masroor FA, Gilde J, Liang J (2018). Vidian nerve schwannoma: a rare skull-base neoplasm presenting with ocular manifestations: a case report and literature review. Perm J.

[11] Xie Y, Yang Q, Xie G, Pang J, Fan Z, Li D (2016). Improved black-blood imaging using DANTE-SPACE for simultaneous carotid and intracranial vessel wall evaluation. Magn Reson Med.

[12] Niepel AL, Steinkellner L, Sokullu F, Hellekes D, Kömürcü F (2019). Long-term follow-up of intracapsular schwannoma excision. Ann Plast Surg.

